# Lesion-to-Liver SUVmax Ratio to Improve the Prognostic Value of the End of Treatment PET/CT in Diffuse Large B-Cell Lymphoma

**DOI:** 10.3390/jcm11195541

**Published:** 2022-09-21

**Authors:** Cristina Ferrari, Antonio Rosario Pisani, Tamara Masi, Giulia Santo, Paolo Mammucci, Dino Rubini, Angela Sardaro, Giuseppe Rubini

**Affiliations:** 1 Nuclear Medicine Unit, Interdisciplinary Department of Medicine, University of Bari ‘Aldo Moro’, 70124 Bari, Italy; 2 Section of Radiology and Radiation Oncology, Interdisciplinary Department of Medicine, University of Bari ‘Aldo Moro’, 7012 Bari, Italy

**Keywords:** DLBCL, PET/CT, Lesion-to-Liver ratio, LLR

## Abstract

Background: Diffuse large B-cell lymphoma (DLBCL) is the most common non-Hodgkin lymphoma worldwide. After first-line therapy, 30–40% of patients relapse or experiment with refractory disease. 18F-FDG PET/CT represents a validated diagnostic tool in post-treatment evaluation of FDG-avid lymphoma, and the Deauville Score (DS), a five-point visual scale, is usually used to assess response. However, the increased number of false positive findings suggested the need to search for new parameters. The aim of this study is to evaluate the prognostic value of End-of-Treatment-PET, comparing DS to the semi-quantitative Lesion-to-Liver ratio (LLR). Methods: newly diagnosed DLBCL patients who underwent 18F-FDG PET/CT were retrospectively analyzed. End-of-Treatment PET findings were assessed first using DS; secondly, assigned the LLR. Results: a total of 105 patients were finally enrolled. ROC analysis showed an LLR of 1.80 as the optimal cutoff value for predicting a disease progression (sensitivity 58%, specificity 95%). Both DS and LLR showed a statistically significant correlation with PFS and OS. LLR resulted in a better diagnostic performance than DS. Conclusions: LLR showed to be a reliable diagnostic method to assess treatment response in DLBCL. The integration of visual and semi-quantitative criteria could help in decision making, improving specificity and PPV.

## 1. Introduction

Diffuse large B-cell lymphoma (DLBCL) represents the most common non-Hodgkin lymphoma (NHL) worldwide. About 60% of patients with DLBCL undergo successful curative first-line chemotherapy, but one-third of patients experiences relapsed or refractory disease [1]. Therefore, identifying no-responders to first-line therapy is essential to guide further treatment strategies [2,3].

Nowadays, 18F-Fluorodeoxyglucose (18F-FDG) positron emission tomography/computed tomography (PET/CT) is recommended at first staging and after the end of therapy in 18F-FDG–avid lymphoma types, such DLBCL [4,5,6,7]. The well-known Lugano response classification based on the 5-point visual scale Deauville Score (DS) is adopted to assess treatment response [8,9]. After the end of first-line therapy, a positive PET/CT scan is usually interpreted as an unfavorable prognostic sign in all DLBCL stages. Remarkably, the prospective Swiss Group for Clinical Cancer Research trial reported that in patients who completed six courses of R-CHOP (rituximab plus combined cyclophosphamide, doxorubicin, vincristine, and prednisone), a lower event-free survival for PET-positive patients than for those with a negative scan (48% vs. 74%) [10,11].

In contrast to the high negative predictive value (NPV) of End-of-Treatment (EoT) PET/CT using the DS (range 78–100%), its positive predictive value (PPV) is generally lower and more variable, ranging from 32% to 100%. This marked PPV variability is probably related to post-therapy inflammatory changes that could affect the interpretation of residual uptake, resulting in an interobserver evaluation variability in scoring DS 3 and 4 [12,13]. Nowadays, in case of persistent doubtful positive sites, a biopsy is performed in routine practice [14]. These findings suggested the need to search for new criteria to overcome the aforementioned DS limitations in NHL. Among semi-quantitative PET parameters tested, some authors are exploring the reliability of the ratio of Lesion-to-Liver maximum standardized uptake value (LLR) as a new threshold to assess PET positivity, but the literature data are still sparse [15,16,17].

This study aims to evaluate the prognostic value of EoT-PET by comparing DS to LLR for progression-free survival (PFS) and overall survival (OS) in patients with DLBCL after first-line immuno-chemotherapy.

## 2. Materials and Methods

### 2.1. Patients

Newly diagnosed DLBCL patients screened with 18F-FDG PET/CT were retrospectively analyzed. The inclusion criteria were: histologically proven DLBCL; age ≥ 18 years; six or eight cycles of rituximab-based standard immuno-chemotherapy; baseline-PET; EoT-PET performed within 12 weeks after the last dose of immuno-chemotherapy; a minimum follow-up period of 12 months. Follow-up was performed considering physical examination, CT scan, or subsequent PET/CT examinations. Events recorded during follow-up were considered as the standard to assess persistent/progressive disease.

Clinical data, including age, gender, Ann Arbor stage, sites of extranodal involvement, and bulky disease (one or more involved sites with a maximum diameter ≥ 10 cm) were collected.

### 2.2. PET/CT Acquisition

PET/CT imaging was performed on a Discovery IQ (GE, Healthcare Technologies, Milwaukee, WI, USA). The field of view and pixel size of the PET images reconstructed for fusion was 70 cm and 2.73 mm, respectively, with a matrix size of 256 × 256. The technical parameters used for CT imaging were: pitch 0.98, gantry rotation speed of 0.5 s/rot, 120 kVp, and modulated tube current of 140 mA. After 6 h of fasting, patients received an intravenous injection of 2.5 MBq/kg 18F-FDG. About 60 min after 18F-FDG administration, CT images were obtained from the skull base to the midthigh. A 3D acquisition mode PET scan for the same longitudinal coverage, 2.5 min per bed position, was performed. CT images were used for attenuation correction, anatomical information, and image interpretation. Image analysis was carried out using a dedicated console (AW Server 4.7, General Electrics, Milwaukee, WI, USA).

Baseline-PET and EoT-PET of each patient were performed with the same PET/CT scanner.

### 2.3. PET/CT Analyses

In total, two nuclear physicians with almost 10 years of experience (C.F. and A.R.P.) evaluated the EoT-PET exams independently and while blinded to patient outcomes. PET results were assessed using first the visual 5-point-scale DS, secondly assigned the LLR.

As usually, the DS includes five risk classes, which are defined as follows: DS 1, no residual uptake; 2, residual uptake not exceeding mediastinal uptake; 3, residual uptake above mediastinal, not exceeding liver uptake; 4, residual uptake moderately above liver uptake; 5, residual uptake markedly above liver uptake and/or new lesions; X, newly emerged uptake unlikely to be related to lymphoma. According to the Lugano classification, patients who scored 1, 2, and 3 at the EoT-PET were considered responders, whereas scores 4 and 5 were assigned to no-responders.

For the LLR-based scale, the maximum standardized uptake value (SUVmax) of the lesion was measured in the most intense focus, if present, on EoT-PET; if no lesion was visible, the SUVmax was conventionally assumed to be equal to the background. In contrast, the SUVmax of the liver was obtained by measuring the SUVmax in a spherical volume of interest (VOI) of 3 cm diameter in the right upper lobe (avoiding the edge and vessels). LLR was then derived.

### 2.4. Statistical Analysis

PFS was defined as the time from the diagnosis to the first evidence of progression or relapse. OS was defined as the time from the diagnosis until the death of any cause. Data were censored if patients were alive with no progression or relapse at the last follow-up. Receiver Operator Characteristic (ROC) curve analyses were performed to determine the optimal cutoff value of LLR. This cutoff value was applied to stratify patients in relation to the presence of disease relapse or progression. Survival analysis was conducted using Kaplan–Meier (K-M) methods, and differences between groups were tested with the log-rank test. The survival analysis was also conducted considering only the sub-group of patients who underwent the R-CHOP chemotherapy scheme to exclude possible interference from different regimens. The agreement between disease relapse/progression assessment established according to LLR and DS (4, 5) was estimated using Cohen’s kappa analysis. The multivariate Cox model was adopted to assess the association between qualitative and semi-quantitative PET parameters and baseline clinical patients’ characteristics. The predictive performance of EoT-PET according to DS and LLR was also calculated.

Statistical analysis was performed using R version 4.1.2 (R Core Team (2021). R: A language and environment for statistical computing. R Foundation for Statistical Computing, Vienna, Austria. URL https://www.R-project.org/). A *p*-value of less than 0.05 indicated statistical significance.

## 3. Results

### 3.1. Patients’ Characteristics

A total of 105 patients with newly diagnosed DLBCL were enrolled. At diagnosis, 46/105 (44%) patients present the IV stage. The median age at diagnosis was 61 years old (range: 18–88), counting 62 males and 43 females. The majority (79%) of patients were treated with 6 or 8 cycles of the R-CHOP chemotherapy regimen. The median time between the last chemotherapy cycle and the EoT-PET was 34 days (range 17–90). The median follow-up duration was 30 months (range 17–113). A total of 43/105 patients progressed or relapsed, and 25 patients died during follow-up. Patient characteristics are shown in Table 1.

### 3.2. Survival Analyses Based on DS vs. LLR

Considering the response assessment based on the DS, 48 (45.7%) patients were assigned DS 1, 5 (4.8%) DS 2, 13 (12.4%) DS 3, 21 (20.0%) DS 4, and 18 (17.1%) DS 5. Applying DS stratification (DS 1–3 response, DS 4–5 no response), DS 4–5 group showed a significantly worse outcome than DS 1–3 (PFS, *p* < 0.001; OS, *p* < 0.001).

**Figure 1:**
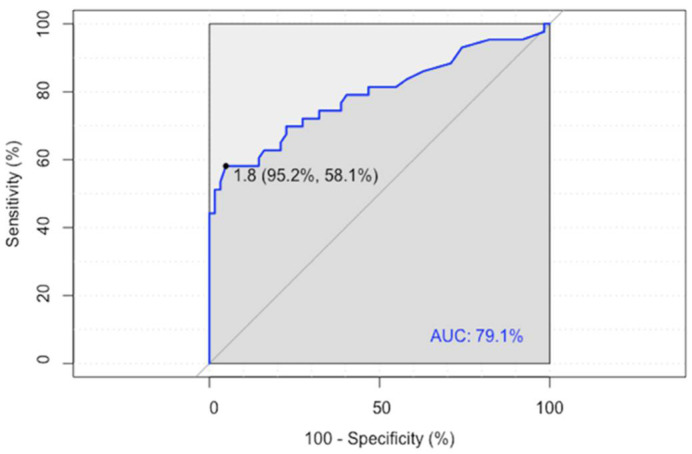
ROC analysis of the Lesion-to-Liver SUVmax ratio (LLR).

ROC analysis showed an LLR of 1.80 as the optimal cutoff value for predicting a disease relapse/progression (sensitivity 58%, specificity 95%), with an area under the curve (AUC) of 0.791 (95% CI 0.696–0.886) (Figure 1).

Applying the optimal LLR cutoff of 1.80 for patients’ stratification, a significantly poorer PFS and OS were observed in patients with higher LLR (*p* < 0.001).

The median follow-up time duration to calculate the Kaplan–Meyer curves for PFS and OS was 25 months (range 6–113) and 30 months (range 17–113), respectively. The estimated 2-years PFS and OS rates were 69% and 82%, while the 3-years PFS and OS rates were 67% and 77%, respectively.

**Figure 2:**
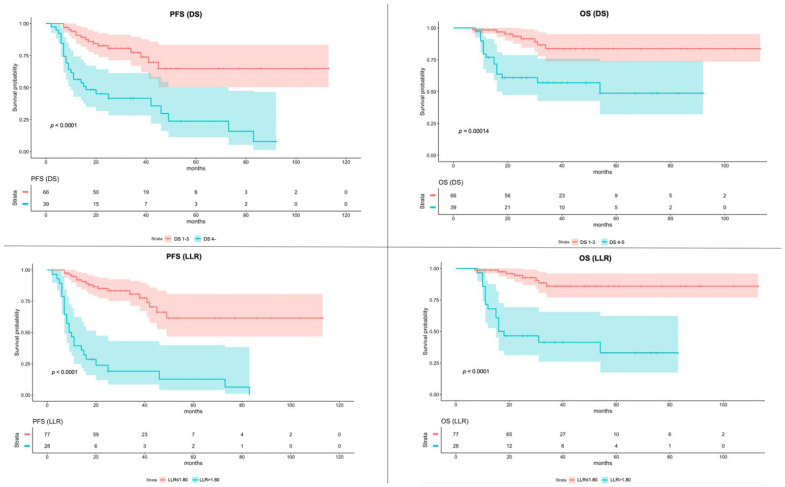
Kaplan–Meier of Progression-Free Survival (PFS) and Overall Survival (OS) according to the Deauville Score (DS) and Lesion-to-Liver SUVmax ratio (LLR). The color bands around the median values of PFS and OS represent the 95% confidence interval.

Figure 2 shows K-M survival curves of PFS and OS of patients dichotomized with DS and LLR, respectively.

**Figure 3:**
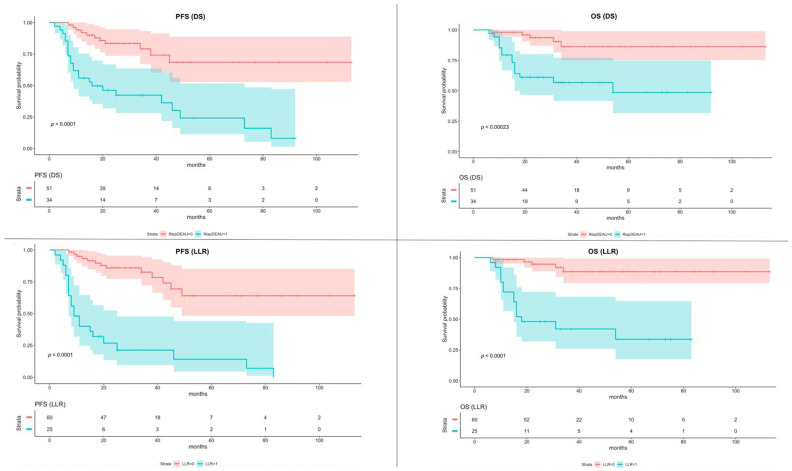
Kaplan–Meier of Progression-Free Survival (PFS) and Overall Survival (OS) according to the Deauville Score (DS) and Lesion-to-Liver SUVmax ratio (LLR) in sub-group of patients underwent R-CHOP scheme. The color bands around the median values of PFS and OS represent the 95% confidence interval.

A sub-analysis was conducted including only patients who underwent the R-CHOP chemotherapy scheme, using, for the LLR evaluation, the same cutoff value of 1.80. The K-M survival curves of PFS and OS showed statistical significance both for DS and LLR. The K-M survival curves of PFS and OS of patients who underwent R-CHOP chemotherapy, dichotomized with DS and LLR, are shown in Figure 3.

Table 2 shows the multivariate analysis results. Only the LLR results were statistically significant for PFS (*p* = 0.00178) but not for OS. In contrast, the DS does not show significance both for PFS and OS.

A good agreement was observed between disease relapse/progression established according to DS and LLR cutoff patients’ stratification (Cohen’s Κ: 0.76, 95% CI: 0.63–0.90, *p* < 0.001). Focusing on disagreement cases (*n* = 11), all patients were considered no-responders according to DS (all DS 4) but presented an LLR ≤ 1.80. Out of all these cases, only 2/11 showed progressive disease during follow-up.

**Figure 4:**
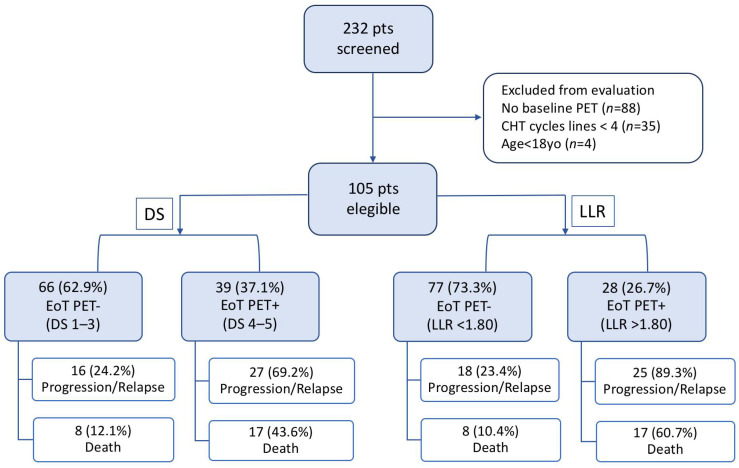
Flow chart of the selected patients’ outcomes screened either with Deauville Score (DS) or Lesion-to-Liver SUVmax ratio (LLR).

The flow chart summarized PET results and outcome of selected patients (Figure 4).

### 3.3. Diagnostic Performance

Compared to DS criteria, LLR using 1.80 as cutoff showed higher specificity (disease progression, LLR vs. DS 95.1% vs. 80.7%; death, LLR vs. DS 86.3% vs. 72.5%), PPV (disease progression, LLR vs. DS 89.3% vs. 69.2%; death, LLR vs. DS 60.7% vs. 43.6%), and accuracy (disease progression, LLR vs. DS 80.0% vs. 73.3%; death, LLR vs. DS 81.9% vs. 71.4%). All diagnostic performance values are detailed in Table 3.

## 4. Discussion

Despite the high rate of responders after first-line chemoimmunotherapy, 30–40% of DLBCL relapse. In the case of primary refractory disease or early relapse, patients showed poor response rates to further treatment lines (26%) and a median OS of 6.3 months [2,18].

Our study aims to assess the prognostic value of EoT-PET through the evaluation of new reliable metabolic parameters that could improve the diagnostic performance of the standardized DS. Notably, the prognostic value of DS in PET-positive patients is under debate due to its low PPV [19,20,21]. Recently, different PET-derived semi-quantitative parameters have been investigated with preliminary study results. Many authors demonstrated that the LLR showed a better interobserver agreement, thanks to the semi-quantitative nature of the score that could outperform the visual analysis. Li et al. conducted a study on 449 newly diagnosed DLBCLs performing a survival analysis based both on DS and LLR. They demonstrated that using 1.83 as the optimal cutoff of LLR, both PFS and OS were significantly different between EoT-PET-positive and EoT-PET-negative patients (both *p* < 0.001) [17]. In another study conducted by Toledano and colleagues, a threshold of 1.4 of LLR was identified as a good prognostic index in a cohort of 180 DLBCL patients both at interim and EoT-PET [22]. In our study, despite the smaller sample size, a similar cutoff value was found as a predictor of prognosis. The cutoff of 1.80 resulted in better predicting progression than the conventional DS criteria. Interestingly, the same cutoff was able to predict outcomes even when the analysis was restricted to a more homogeneous cohort of patients who underwent the R-CHOP chemotherapy regimen.

Toledano et al. also confirmed that the prognosis accuracy using LLR allowed a significant increase in PPV compared to DS and then a decreased number of false positives [22]. In our sample, the LLR-based criterion displayed a PPV of 89.3% in predicting progression/relapse and 60.7% for survival, exceeding DS and confirming the existent data.

The routine use of LLR could be supported by some important advantages. First, the independence from the administered activity and body weight, overcoming the limits of other semi-quantitative parameters such as ΔSUVmax. Moreover, it allows the conversion of a visual qualitative scale (e.g., DS) to a continuous semi-quantitative one [23,24]. In addition, the definition of a semi-quantitative cutoff value simplifies the dichotomization of responder vs. no-responder patients at EoT-PET; however, caution should be made in the assessment of liver disease and diabetes, factors that may influence liver metabolism. In our sample, no patient presented a history of hepatic disease. Regarding diabetes, the main issue that should be considered is the blood glucose level at the moment of FDG administration. Dietetic and therapeutic protocols must be adopted in order to decrease at the minimum level the risk of hyperglycemia. These considerations pointed out the need for protocol standardization in order to increase reproducibility, avoiding potential pitfalls of reference region [25,26].

**Figure 5:**
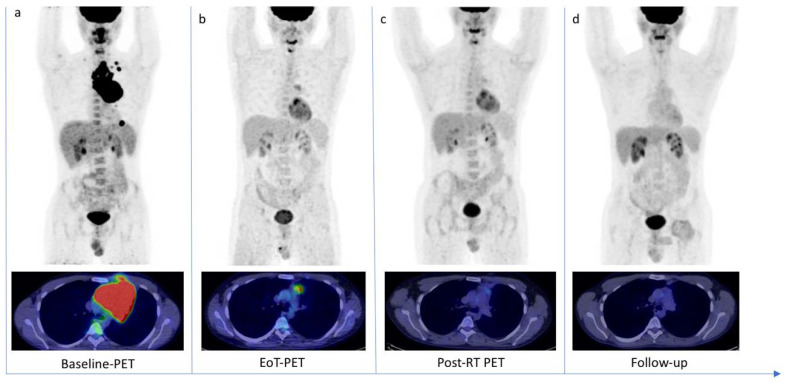
18F-FDG PET/CT scans of a 30-year-old patient with diffuse large B cell Lymphoma. (**a**) Baseline-PET showed an abnormal hypermetabolic mediastinal tumoral lesion (SUVmax 34.3) with supradiaphragmatic lymph nodes involvement (SUVmax 28.1). The patient underwent six cycles of chemotherapy. (**b**) The End-of-Treatment PET (EoT-PET) showed a partial metabolic response, with consistent morpho-metabolic disease reduction but persistent uptake in mediastinum (SUVmax 4.8): Deauville Score 4 was assigned and a Lesion-to-Liver Ratio of 1.45 was calculated. Patient underwent radiotherapy. (**c**) Post-radiotherapy PET (Post-RT PET) demonstrated a further reduction in tumor size and glucose metabolism (SUVmax 2.7). (**d**) After 10 months, a follow-up scan demonstrated a complete metabolic response (OS 25 months).

Interestingly, in our study, we found that all discordant cases belong to DS 4. In contrast, DS 4–5 were normally defined as no-responders at EoT-PET; our analysis showed that DS 4 with an LLR < 1.80 had a more favorable outcome (Figure 5).

These results are consistent with literature data that suggest not to unconditionally classify DS 4 as PET positive, performing a further reclassification using an LLR cutoff value [17,21].

This study was limited by the retrospective and monocentric nature, the relatively small sample size compared to the literature, and the heterogeneity of first-line treatment modalities.

## 5. Conclusions

To conclude, the semi-quantitative assessment of treatment response at EoT-PET, based on LLR, could have higher reliability than visual analysis to predict outcomes. The integration of both criteria could help the making-decision assessment in DLBCL patients, improving specificity and PPV with an impact on PFS and OS. Further prospective studies are warranted to confirm these data.

## Figures and Tables

**Table 1:** Patient characteristics.

Total Patients	105 (100%)
Gender	
Male	62 (59%)
Female	43 (41%)
Age at diagnosis	
Median (range)	61 (18–88)
Follow-up	
Median (range)	30 (17–113)
Chemotherapy	
R-CHOP	85 (81%)
R-COMP	12 (11%)
EPOCH-R	5 (5%)
R-MACOP-B	3 (3%)
Ann Arbor Stage	
Stage I-II	36 (34%)
Stage III-IV	69 (66%)
Bulky	
Yes	16 (15%)
No	89 (85%)
Extranodal Involvement	
Yes	55 (52%)
No	50 (48%)

Legend: *R-CHOP*, rituximab plus combined cyclophosphamide, doxorubicin, vincristine, and prednisone; *R-COMP*, rituximab, cyclophosphamide, non-pegylated liposomal doxorubicin, vincristine, and prednisone; *EPOCH-R*, etoposide, prednisone, vincristine, cyclophosphamide, doxorubicin, and rituximab; *R-MACOP-B*, rituximab, methotrexate, leucovorin, doxorubicin, cyclophosphamide, vincristine, prednisone, bleomycin.

**Table 2:** The multivariate analysis for PFS and OS includes qualitative and semi-quantitative PET parameters and baseline clinical patients’ characteristics.

Variable	PFS		OS	
	*p* Value	HR (95% CI for exp(b))	*p* Value	HR (95% CI for exp(b))
**Age**	0.969	0.999 (0.980–1.020)	0.0154	1.040 × 10^0^ (1.0075–1.073)
**Diabetes**	0.529	0.732 (0.277–1.935)	0.2897	1.696 × 10^0^ (0.6380–4.507)
**Sex**	0.757	0.898 (0.455–1.773)	0.0421	3.400 × 10^−1^ (0.1202–0.962)
**Stage**	0.828	0.917 (0.419–2.005)	0.6733	8.056 × 10^−1^ (0.2948–2.201)
**Extranodal sites**	0.753	0.894 (0.446–1.795)	0.1930	1.923 × 10^0^ (0.7186–5.144)
**Bulky disease**	0.734	0.851 (0.336–2.154)	0.2572	4.707 × 10^−1^ (0.1278–1.733)
**DS**	0.574	0.650 (0.145–2.920)	0.9979	1.413 × 10^−8^ (0–inf)
**LLR**	0.00178	10.939 (2.4401–49.037)	0.9976	7.581 × 10^8^ (0–inf)

Legend: *PFS*, progression-free survival; *OS*, overall survival; *DS*, Deauville Score; *LLR*, Lesion-to-Liver ratio.

**Table 3:** The predictive performance of EoT-PET according to DS and LLR.

		Sensitivity	Specificity	PPV	NPV	Accuracy
**Progression/Relapse**	**DS**	62.8	80.7	69.2	75.8	73.3
	**LLR**	58.1	95.2	89.3	76.6	80.0
**Survival**	**DS**	68.0	72.5	43.6	87.9	71.4
	**LLR**	68.0	86.3	60.7	89.6	81.9

Legend: *PPV*, Positive Predictive Value; *NPV*, Negative Predictive Value; *PFS*, Progression-Free Survival; *OS*, Overall Survival; *DS*, Deauville Score; *LLR*, Lesion-to-Liver SUVmax ratio.

## Data Availability

The data presented in this study are available on request from the corresponding author.
